# A real 3D measurement technique for the tibial slope: differentiation between different articular surfaces and comparison to radiographic slope measurement

**DOI:** 10.1186/s12891-020-03657-9

**Published:** 2020-09-26

**Authors:** Armando Hoch, Lukas Jud, Tabitha Roth, Lazaros Vlachopoulos, Philipp Fürnstahl, Sandro F. Fucentese

**Affiliations:** 1 Department of Orthopaedics, Balgrist University Hospital, Forchstrasse 340, 8008 Zurich, Switzerland; 2 Research in Orthopaedic Computer Science, Balgrist University Hospital, University of Zurich, Zurich, Switzerland

**Keywords:** Tibial slope, 3D measurement, Articular surface

## Abstract

**Background:**

The tibial slope plays an important role in knee surgery. However, standard radiographic measurement techniques have a low reproducibility and do not allow differentiation between medial and lateral articular surfaces. Despite availability of three-dimensional imaging, so far, no real 3D measurement technique was introduced and compared to radiographic measurement, which were the purposes of this study.

**Methods:**

Computed tomography scans of 54 knees in 51 patients (41 males and 10 females) with a mean age of 46 years (range 22–67 years) were included. A novel 3D measurement technique was applied by two readers to measure the tibial slope of medial and lateral tibial plateau and rim. A statistical analysis was conducted to determine the intraclass correlation coefficient (ICC) for the new technique and compare it to a standard radiographic measurement.

**Results:**

The mean 3D tibial slope for the medial plateau and rim was 7.4° and 7.6°, for the lateral plateau and rim 7.5° and 8.1°, respectively. The mean radiographic slope was 6.0°. Statistical analysis showed an ICC between both readers of 0.909, 0.987, 0.918, 0.893, for the 3D measurement of medial plateau, medial rim, lateral plateau and lateral rim, respectively, whereas the radiographic technique showed an ICC of 0.733.

**Conclusions:**

The proposed novel measurement technique shows a high intraclass agreement and offers an applicable opportunity to assess the tibial slope three-dimensionally. Furthermore, the medial and lateral articular surfaces can be measured separately and one can differentiate the slope from the plateau and from the rim. As three-dimensional planning becomes successively more important, our measurement technique might deliver a useful supplement to the standard radiographic assessment in slope related knee surgery.

**Level of evidence:**

Level III, diagnostic study.

## Highlights

- The tibial slope plays an important role in knee surgery planning
- Available measurement techniques show a low intraclass correlation
- So far, no real 3D measurement technique was introduced
- Our novel 3D measurement technique showed an excellent intraclass correlation

## Background

The inclination of the proximal articular surface of the tibia was first described and introduced as tibial slope in an anatomical study in 1951 [1]. As total knee replacement was gaining popularity, the importance of the tibial slope in sagittal plane balancing was recognized [2–4]. The tibial slope was depicted as an important factor influencing postoperative knee flexion and stability after total and unicompartmental knee replacement and various recommendations were made to restore or increase the native slope to optimize range of motion [5–10]. Furthermore, an increased tibial slope was recognized as a risk factor for anterior cruciate ligament (ACL) rupture in native knees [11–14], whereas a decreased tibial slope could be identified as a risk factor for posterior cruciate ligament (PCL) injury [15]. These findings were used to reduce the forces acting on ACL-grafts and to address instability in ACL-deficient knees by reducing the tibial slope when performing a high tibial osteotomy (HTO) [15–18].

Measurement of the tibial slope has been discussed controversially. Various two-dimensional measurement techniques in lateral radiographs of the knee are available [19–22]. There is lack of clarity regarding the accuracy of these measurements: On the one hand the anatomical tibial axis cannot reliably be determined on most lateral knee radiographs and, on the other hand, one cannot differentiate between the medial and the lateral articular surface. These factors lead to an unsatisfying intraclass correlation [23–26]. Therefore, different three-dimensional techniques have been proposed [25–32]. To our knowledge, there is no measurement technique which considers the three-dimensional anatomical tibial axis and the volumetric constitution of the proximal tibia to differentiate between medial and lateral plateau and rim in a three-dimensional model.

The first purpose of this study was to introduce such a technique. Furthermore, no comparison between three-dimensionally assessed tibial slope and standard radiographic assessment has been conducted, which was the second purpose of our study. The hypothesis was that such a 3D technique can be introduced for the measurement of the tibial slope and that it has a high robustness, superior to the standard radiographic measurement.

## Methods

### Patient selection

We retrospectively identified 82 consecutive patients who received a computed tomography (CT) scan of one or both native knees, including the proximal 15 cm of the tibia. In all patients a lateral view radiograph of the investigated knees was available. All CT scans were performed at our institution from 11/2016 to 01/2019. These patients were all scheduled for an osteotomy navigated by patient specific instruments for different reasons. Nineteen patients who underwent a correction of the tibial slope were excluded subsequently in order to obtain a collective without slope-related problems. Two patients were additionally excluded because a slope-related problem could not be ruled out. Two patients with a posttraumatic status, two patients with prior osseous knee surgery, five patients with dysplasia and one patient with osteogenesis imperfecta were excluded from this study to exclude pathologically altered bony anatomy. Finally, we included CT scans and radiographs of 54 knees from 51 patients (41 males and 10 females) with a mean age of 46 years (range 22–67 years) and a mean BMI of 30.4 kg/m^2^ (range 20.4–41.2 kg/m^2^). They were all planned for HTO for unicompartmental osteoarthritis, whereas the radiographic degree of osteoarthritis was mild (Kellgren and Lawrence grade I or II) in all cases. The local ethical committee approved this study (Zurich Cantonal Ethics Commission, 2018–02242) and all patients gave their informed consent for the use of their data for research purposes.

### CT examinations

All CT scans were performed at our institution, using Siemens Definition AS® or Somatom Edge CT® scanners. Slice thickness was 1.0 mm with an in-plane resolution (x-y) of 0.4 × 0.4 mm.

The CT scans were segmented using the global thresholding and region growing functionality of a standard segmentation software (Mimics Medical, Materialise NV, Leuven, Belgium) in order to generate 3D bone models [33–35].

### 3D slope measurement technique

**Fig. 1:**
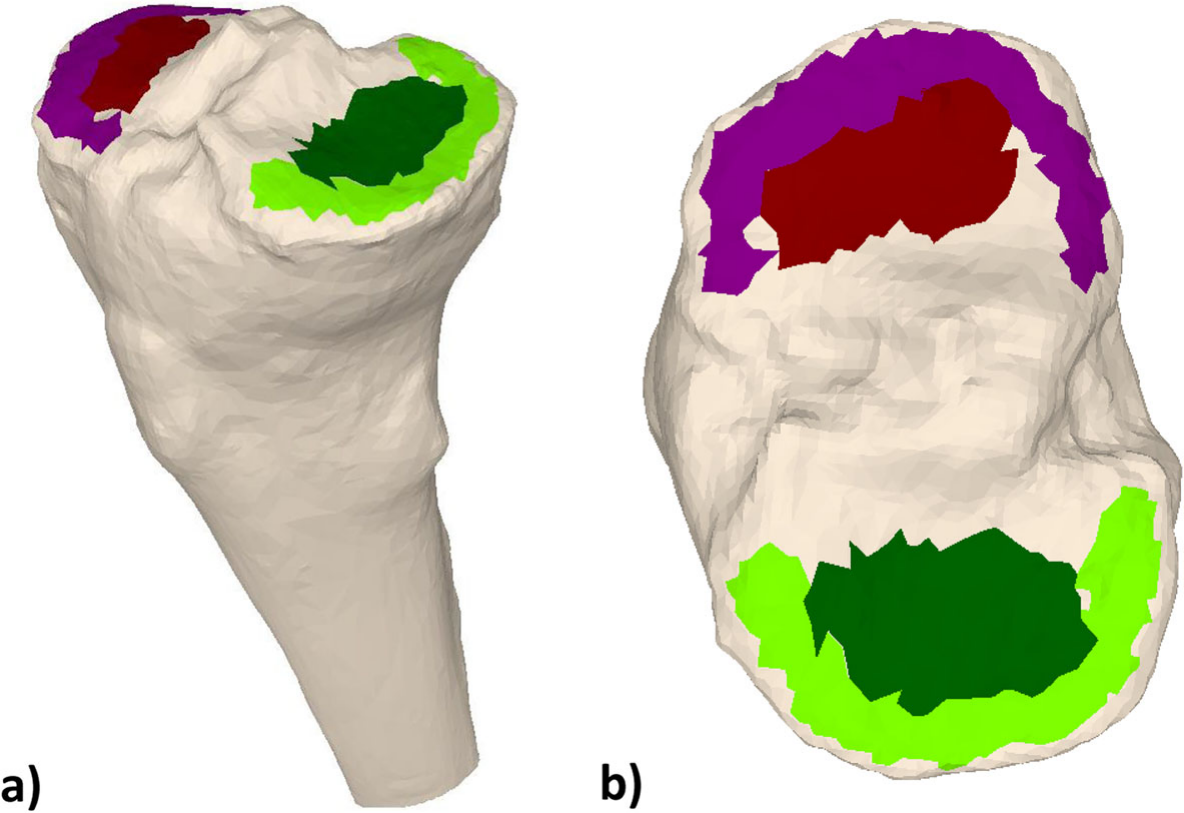
Four different brushed parts of the articular surface: Medial plateau (dark green), medial rim (bright green), lateral plateau (dark red), lateral rim (purple) in relation to the proximal tibia (beige) in a view from superomedial **a**) and above **b**)

**Fig. 2:**
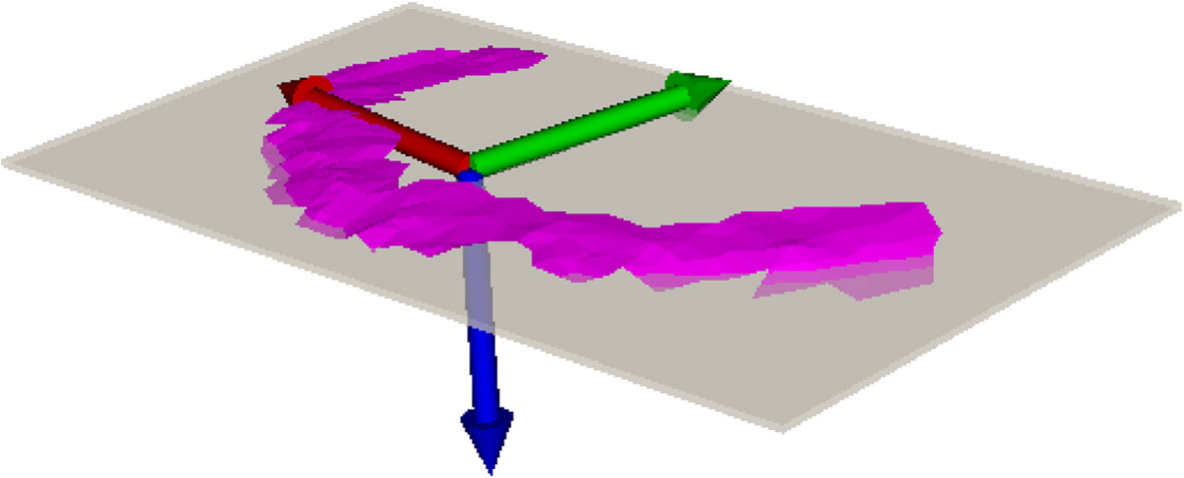
Example for a brush of a lateral rim of the tibia plateau (pink) with the plane (transparent grey) with a minimal squared distance to each of the points of the surface model. Additionally the coordinate system defining the tibial slope normal (z-axis, blue) of this specific model.

Four different parts of the articular surface of the 3D bone models were subsequently marked with a brushing tool (3-matic Medical, Materialise NV, Leuven, Belgium): The medial plateau, the medial rim, the lateral plateau and the lateral rim. These parts are depicted in Fig. 1. They were brushed by the respective investigator. The rim was defined as the outermost, elevated portion of the joint surface. The plateau was defined as the concavity of the articular surface typically opposite the femoral condyle. The intercondylar region was not integrated into the brushing. Using our in-house planning software (CASPA, Balgrist CARD, Zurich, Switzerland), principal component analysis (PCA) was applied to each of the four surface models, creating a plane with a minimal squared distance to each of the points of the surface models [36] (Fig. 2).

**Fig. 3:**
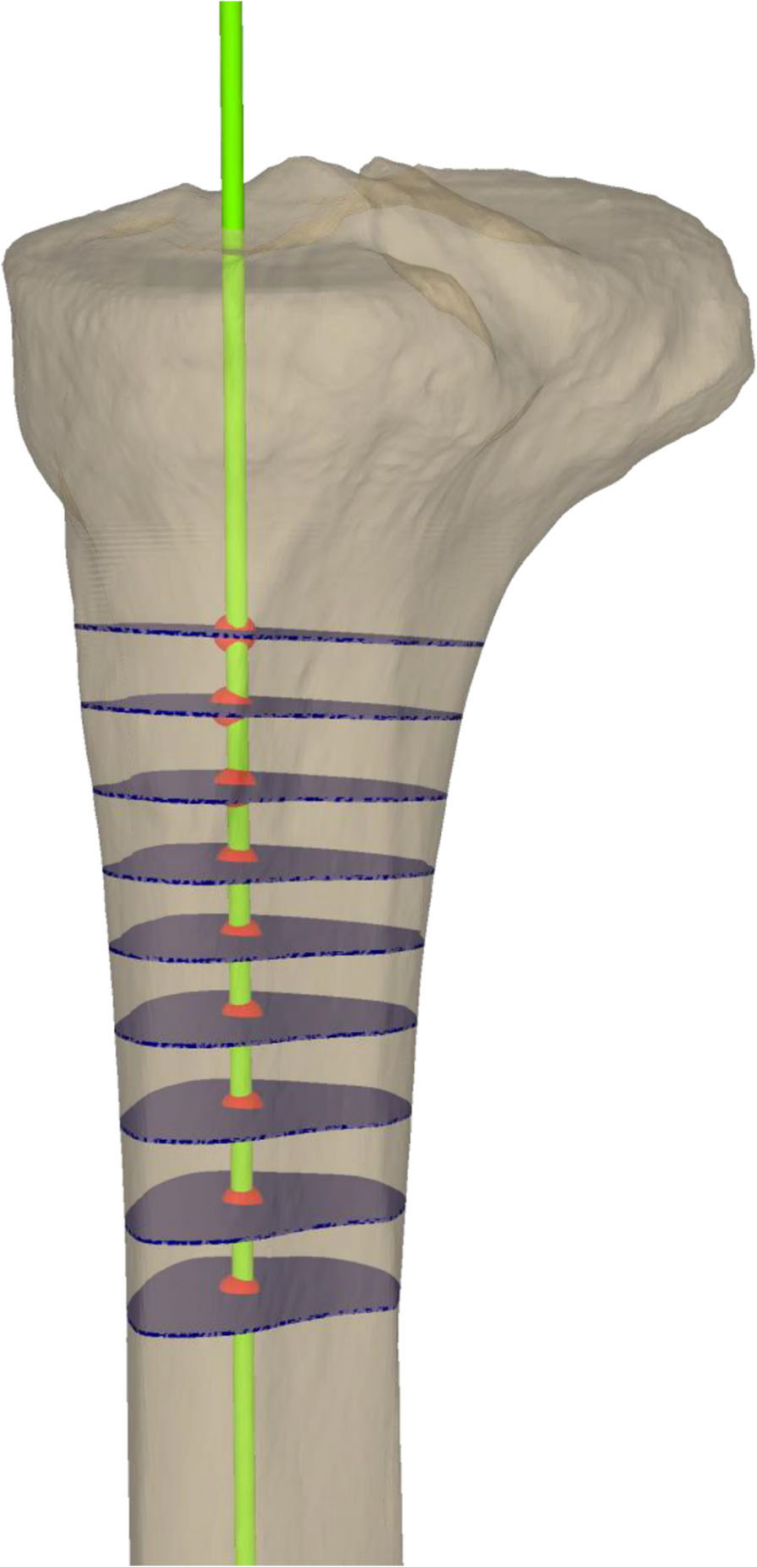
Anatomical axis (bright green) of the tibia (transparent beige), defined by the center points (red) of all cross sections (transparent dark blue)

For the anatomical axis, an axial cross section of the tibial shaft was automatically generated 15 cm distal from the articular knee surface. This procedure was repeated in 5 mm steps proximal from the first cross section, until the cross-section area exceeded a threshold (1.6-fold of the first cross section area). The anatomical axis was then fitted using a least square approach, minimizing the distance to the center points of all cross sections (Fig. 3) [36]. The tibial slope was defined as the angle between the anatomical axis and the tibial slope normal, projected to the sagittal plane. The sagittal plane was defined according to a previously published method [37]. This was done for all aforementioned four articular surface parts. These calculations were made using MATLAB R 2019a (The MathWorks Inc., Natick, Massachusetts, USA).

**Fig. 4:**
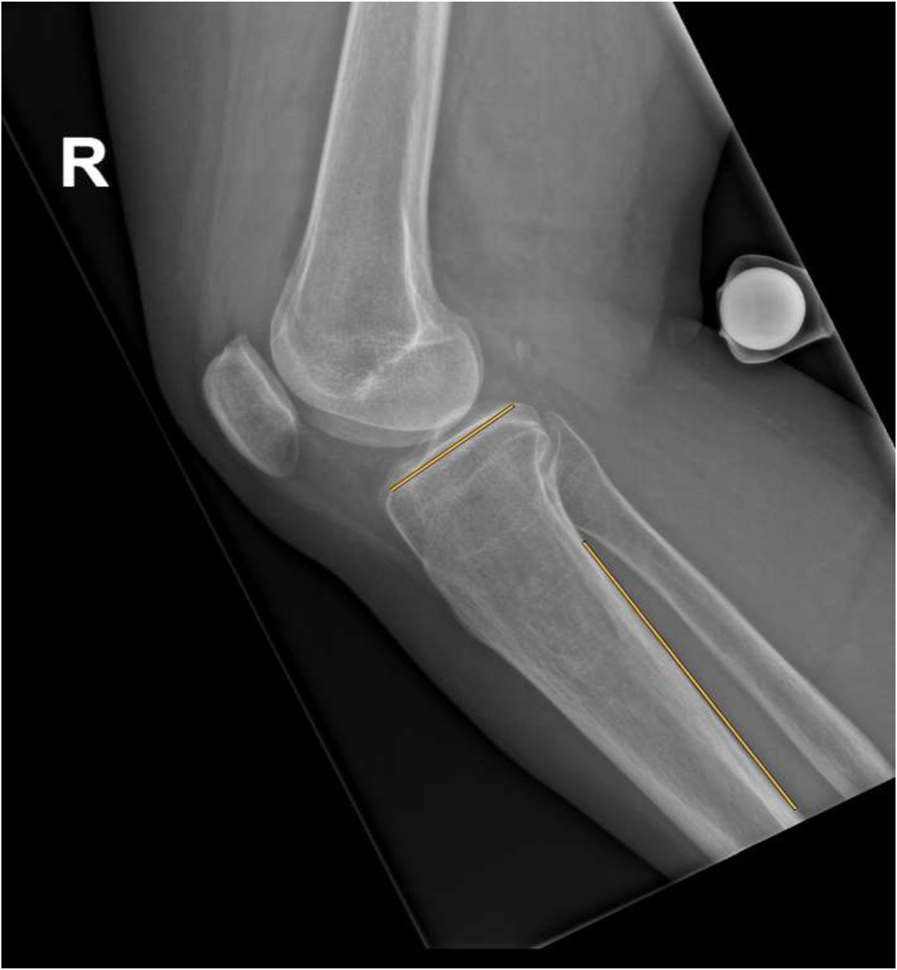
Lateral view radiograph of a right knee with the two lines (orange) for conventional measurement of the posterior tibial slope

On conventional 2D radiographs, we assessed the posterior tibial slope, using the posterior tibial cortex line, which is, to our knowledge, the most frequently applied technique (Fig. 4) [19–22]. The slope was measured as the angle between a line drawn along the posterior tibial cortex and a line adjacent to the horizontally arranged zone of highest radiodensity on the uppermost part of the tibial head. All measurements were performed by two readers (A. H., L. J.).

### Statistical analysis

All data were documented with the REDCap software (Research Electronic Data Capture, Vanderbilt University, Nashville, Tennessee, USA). Statistical analyses, conducted with SPSS (IBM SPSS Statistics 25, IBM, Armonk, New York, USA), were used to provide descriptive data as mean and standard deviation (SD). The intraclass correlation coefficients for the different parameters were calculated based on a two-way random effects model assessing absolute agreement of single measures between readers.

## Results

The mean 3D tibial slope for the medial plateau and rim was 7.4° and 7.6°, for the lateral plateau and rim 7.5° and 8.1°, respectively. The mean radiographic slope was 6.0°. Statistical analysis showed an ICC between both readers of 0.909, 0.987, 0.918, 0.893, for the 3D measurement of medial plateau, medial rim, lateral plateau and lateral rim, respectively, whereas the radiographic technique showed an ICC of 0.733 (Table 1). There were no significant differences between the medial and lateral compartments, nor between the rim and plateau.

**Table 1:** Slope measurements and Intraclass correlation coefficients

reader	medial plateau	medial rim	lateral plateau	lateral rim	radiograph (PTS)
1	**7.5°**	**7.6°**	**7.7°**	**8.2°**	**6.0°**
	SD 4.72°, range − 2.0° – 17.3°	SD 3.91°, range − 1.7° – 14.6°	SD 4.46°, range − 2.3° – 15.1°	SD 2.98°, range 2.2° – 14.6°	SD 2.90°, range − 0.7° – 11.9°
2	**7.3°**	**7.6°**	**7.4°**	**8.0°**	**5.9°**
	SD 3.45°, range − 0.9° – 15.0°	SD 3.80°, range − 1.1° – 14.8°	SD 3.45°, range − 0.1° – 13.2°	SD 3.09°, range 1.1° – 14.7°	SD 3.00°, range − 4.1° – 11.8°
ICC	**0.909**	**0.987**	**0.918**	**0.893**	**0.733**
	95% CI, 0.849; 0.946	95% CI, 0.977; 0.992	95% CI, 0.860; 0.952	95% CI, 0.823; 0.936	95% CI, 0.581; 0.836

*PTS* Posterior tibial slope, *SD* Standard deviation, *CI* Confidence interval, *ICC* Intraclass correlation coefficient

## Discussion

A measurement technique which considers the three-dimensional anatomical tibial axis and the volumetric constitution of the proximal tibia to differentiate between medial and lateral plateau and rim in a three-dimensional model is lacking. The purposes of our study were the introduction of such a technique and its comparison with standard radiographic measurement techniques.

The most important finding of this study is that the proposed novel three-dimensional measurement technique showed a mainly excellent intraclass correlation [38]. Furthermore, the technique is capable of assessing the slope for the lateral and medial plateau and rim separately. We think this is of high importance since from a kinematic point of view the plateau might play a central role as it - and not the rim - contains the weight bearing area [39–41]. Therefore, we propose, that for evaluation of the tibial slope, values derived from the plateaus should be used. In addition, it could be used in special situations, where a differentiation is needed (e.g. posttraumatic situations after tibial plateau fractures or for intra-articular corrective osteotomies). As expected, the intraclass correlation for the 2D measurement technique was low and therefore this technique was clearly inferior to the 3D measurement technique. Although not in the focus of our study, we have compared the different 3D parameters with the 2D measurement results. The interclass correlation coefficients comparing 3D parameters with standard radiographic measurement of the posterior tibial slope were low. The radiographic measurement doesn’t seem to be reflected by one of the different three-dimensional slope measurements. This is in line with the assumption that neither medial nor lateral structures can safely be differentiated in lateral knee radiographs.

Since three-dimensional imaging has become standard in preoperative assessment, several studies were conducted to describe techniques for tibial slope measurements based on these images. Hudek et al. used sagittal MR examinations to measure the medial and the lateral tibial slope separately [25]. They defined the anatomical tibial axis in one single MR slice through the center of the knee in which only the very proximal tibia was available. For the slope measurement a tangent connecting the uppermost anterior and posterior cortex edges of the medial and lateral plateau was drawn. Their proposed assessment of the tibial axis seems questionable because it is dependent from the length of the tibia in the determined sagittal plane. Furthermore, only the rim, and as such not the weight bearing part of the knee, was used for slope determination. In their study intraclass correlation coefficient was 0.77.

Haddad et al. investigated the tibial slope in a larger collective of 143 patients with a new technique for slope assessment in MRI [26]. They also used single cross-sectional slices to determine the tibial slope but in a more reproducible fashion compared to Hudek et al. which consolidated in a higher intraclass correlation coefficient of 0.84. Saxena et al. published an additional measurement technique in 2016 [27]. They were able to evade the problem of the dependency of the sagittal reconstruction described in the work of Hudek et al. by coordinating axial, coronal and sagittal plane. Nevertheless, again the uppermost cortical points were chosen to determinate the slope. The evaluation of reproducibility was not evaluated in their study and therefore an intraclass correlation coefficient was not provided. Amerinatanzi et al. published the first technique using points on the surface of a proximal tibia model based on an MRI [29]. They used multiple points in 2 mm increment on the medial and lateral rim for tibial slope calculation. The intraclass correlation coefficient was 0.909 for the medial and 0.968 for the lateral rim. For calculation of tibial anatomical axis, they used the most proximal two centimeters of the tibia. Their measurements were made on one single individual. One year later an automated version of the same measurement technique was published by the same authors [30]. This time they investigated their technique on nine individuals. Furthermore, they made an adaption that provided information about the slope in areas of kinematic interest, which was defined by the authors as the deepest depressions of the medial and lateral proximal tibial surfaces. Within these areas, again projected points on the rim were used for measurement. Amirtharaj et al. proposed another technique based on CT scans [31]. Their technique contained an automated measurement also using multiple points in 3 degrees increment around a center in the middle of the tibial plateau situated on the uppermost part of the rim. Three cadaveric knees were used. Ho et al. used data from 100 CT scans to develop a 3D measurement technique [32]. For slope measurement the lateral and the medial articular surfaces of the proximal tibia containing plateau and rim were used. Nevertheless, only a smaller part of the collective was measured by a second reader and no intraclass correlation coefficient for the whole collective was provided. None of the mentioned studies compared the three-dimensional slope to standard radiographic slopes.

A limitation of our study is the study population of patients who were designated for a high tibial osteotomy which could suggest a preexisting bony condition. An influence of this condition on the tibial slope is conceptually unlikely. Patients with severe bony defects were not found in our collective, as they would not qualify for HTO. Furthermore, a preexisting pathological slope in our collective as reason for unicompartmental osteoarthritis cannot be excluded but is highly unlikely since the collective shows a mean slope comparable to previous data [4, 21, 23, 24].

Another limitation is the fact that CT scans are not a standard diagnostic tool even in potentially slope related problems and segmentation is time-consuming and requires specific knowledge which is not always accessible. Since CT scans are progressively improved in terms of dose reduction and our measurement technique could be implemented in a computer program, a clinically suitable solution is imaginable [42]. Furthermore, MRI technique is also evolving and new protocols that make segmentation easier will be available. Our method could then also be applied to models derived from MRI data. However, 3D planning and the use of patient specific instruments has gained importance in the last decade and with further development we believe that it will be even more used in the future [43]. Intra-reader ICC was not performed due to the highly standardized definition of the surfaces and that the mainly automatized measurement procedure.

## Conclusion

The proposed novel measurement technique shows a high intraclass agreement and offers an applicable opportunity to assess the tibial slope three-dimensionally. Furthermore, the medial and lateral articular surfaces can be measured separately and one can differentiate the slope from the plateau and from the rim. As three-dimensional planning becomes successively more important, our measurement technique might deliver a useful supplement to the standard radiographic assessment in slope related knee surgery.

## Data Availability

The datasets used and analyzed during the current study are available from the corresponding author on reasonable request.
